# “*There are lots of new faces this year… I’m not entirely sure when I became one of the old ones*”: a psycho-ethnography of the self at #PoWESconf five years in

**DOI:** 10.3389/fpsyg.2023.1280940

**Published:** 2024-01-08

**Authors:** Sergio A. Silverio

**Affiliations:** ^1^Department of Women and Children’s Health, School of Life Course and Population Sciences, King’s College London, London, United Kingdom; ^2^School of Psychology, Faculty of Health, Liverpool John Moores University, Liverpool, United Kingdom

**Keywords:** conferences, feminist praxis, positioning the self, PoWES, psycho-ethnography, scholarship

## Abstract

Conferences have been discussed as spaces for academic work to extend beyond the confines of one’s institution, fostering environments of collaborative working, learning, and social bond-making. The British Psychological Society’s Psychology of Women and Equalities Section hosts an annual conference, attended by feminist scholars from around the world. Drawing on auto-ethnography and psycho-biography, this paper presents a ‘psycho-ethnography of the self’ with reflections centred on: ‘Scholarship’, ‘Feminist Praxis’; ‘(Safe) Academic Spaces’; and ‘Positioning the Self’. This article contributes to a small, but growing body of literature critically reflecting on conferences as spaces for personal and professional development and academic growth.

## 1 Introduction

Conferences offer a brief and unique ‘escape’ from the commutes, stuffy offices, and hours of gazing at dual computer screens most academics endure on a daily basis. They become spaces where colleagues and collaborators from different institutions – and even different countries and disciplines – come together to meet in person (sometimes for the first time, despite having worked and, dare I say, even published together beforehand), plan future research, solicit feedback prior to submitting papers for publication, meet potential new collaborators, and in doing so often form strong professional and personal bonds with one another. There is something quite formulaic about conferences. They are, of course, a ‘performance’ in every sense of the word: Academics usually go armed with their most recent work and give it an airing to their peers and their competitors with feedback being expected – be it positive, negative, or on occasion hurtful (see Bell and King, 2010; Ford and Harding, 2010). Thus, conferences can be described in a Butlerian way whereby the performance is socially constructed and adaptive (Butler, 1988) or in-line with Goffman’s (1959) theory of performances having a ‘front stage’ where the performers are ‘on’ (i.e., the conference talks and times for networking) and a ‘back stage’ where the performers are ‘off’ (i.e., the spaces in which delegates are alone and reflect on their performance). Nonetheless, there is an importance placed on conferences by academics, due not only to their scholarly nature to facilitate and foster new research, but also because of their ability to bring together academics with similar outlooks and research areas and facilitate cross-institutional and increasingly, cross-disciplinary collaborations, networking, mentorship, and if you are lucky enough, even friendship (see Ford and Harding, 2008; Mair and Frew, 2018).

Many academics will recognise the importance of conferences for academic engagement which goes wider than colleagues with whom they share offices and the students they see each year on the courses to which they contribute. Though in the present era of academic austerity, there is evidence to suggest researchers are becoming more discerning over their choice in conference attendance (see Edelheim et al., 2018). Some conferences are more mechanical than others – whereby they follow a routine, and often long-recycled style of welcome, parallel streams, poster galleries, networking sessions over (cheap) wine and mealtimes, and a closing keynote (see Ford and Harding, 2008). As McCulloch (2018); p. 53 wryly quips: “*No matter how many catastrophes the organisers think have occurred during the course of a conference, as long as the conference venue does not explode or self-combust, the delegates will assume that everything happened as it was planned*.’’ Conferences often have an organised chaos feel about them, but somehow The British Psychological Society [BPS] Psychology of Women and Equalities Section [PoWES] annual conference manages to contain all the so-called required elements, but is different in a very positive way. The conference – now with the more recent additions of parallel workshops and engaging symposia – is a three-day event which simply does not have that same mechanical feel to it, as it is more relaxed, with an emphasis on inspiring conversation and debate, rather than harsh critiques of work presented, and so delegates place an importance on critical perspectives and a global, intersectional, feminist agenda. The PoWES annual conference is nurturing, wholesome, and supportive. It is just as welcoming to new members, as it is to those who have been in attendance since its inception. These feelings have been shared widely by the members and delegate (see Capdevila et al., 2019; Donnelly et al., 2022), but the secret behind as to why this is, remains unknown or perhaps simply un-nameable (see also Bowes-Catton, 2023). A sound guess would be that it is the type of members who engage in a feminist community such as PoWES, which make the conference feel so different to most others. What the PoWES conference offers is greater than a sharing of academic knowledge, but rather a vital space for critical reflection, mentoring, and both personal and professional growth. Networking is also important, but it follows a model which is not based on simply how useful someone can be to the development of your own research, but rather is based on common interest, camaraderie, and on building strong systems of support. It is relaxed, over informal meals, walks in the grounds, and open mics, and not over black-tie dinners, with grand speeches from ‘the pale, male, and stale’ of academia. These supportive networks are there to ensure all those who are involved are provided with the scaffolding they need to continue in their academic careers; the advice they require to overcome difficulties and challenges in their current circumstances; and to re-energise ideas to ensure that within academia, Psychology, and in all aspects of our lives, the feminist agenda for equity in rights and freedoms are pushed to make real, tangible, national and global change.

Conferences themselves require a degree of psychological labour to be undertaken whereby delegates are in essence, complicit in the construction of a grand performance (i.e., attending, presenting, and networking), where they each have a role to enact – a role which may change depending on their notoriety amongst the(ir) academic community, the stage of their academic career they are in, and also their role at the conference (i.e., as conference Chair, as a keynote, as a session chair or symposium convener, as a speaker, or as an attendee). There is an etiquette to conferences which, although abided by, can be psychologically taxing (see Ford and Harding, 2008; Edelheim et al., 2018; Mair and Frew, 2018). Scholarship on the experiences people have at conferences remains fairly limited and analyses of delegates’ expectations, attendance, and the subsequent outcomes of conference attendance is a relatively unexplored part of the global academic endeavour. Though where it does exist it focuses on their actual utility and potential within academia (Nicolson, 2017; Benozzo et al., 2019); knowledge production and academic impact (Shalom, 1993; Rowley-Jolivet, 2004; Gross and Fleming, 2011; Ioannidis, 2012; de Leon and McQuillin, 2018); academia-based anxieties (Ford and Harding, 2008; Bhandari, 2017); and body language exhibited by delegates (Bell and King, 2010). Literature which uses a gendered lens to analyse academic conferences is generally lacking, and therefore there is limited scholarship available from the last few decades. Published material which does exist covers the discrimination women face in attending (Eden, 2016) or whilst at conferences (see Bell, 1987; Ford and Harding, 2010); or trying to unpack gender (im)balances at them (see New and Fleetwood, 2006; Jankowski, 2016; Mair and Frew, 2018).

This paper, therefore, goes someway to contribute to this small, but growing body of literature. In order to do this, the article presents a ‘psycho-ethnography of the self’ – a mixture of an auto-ethnography and a psycho-biography – of my fifth time attending The BPS PoWES annual conference. The article explores how I have perceived my role within the conference delegation change from a ‘new face’ when I first attended as an undergraduate Psychology student, to having the perception I have now come to be one of the ‘old faces’, having at the time this article was written and subsequently updated for submission, attended the annual conference consecutively from 2015 to 2023.

## 2 Methodology

This paper presents, perhaps, an unusual take on an ethnographic approach, whereby the methodology employed is reminiscent of both auto-ethnography and psycho-biography, in what I, as the author of this paper, have termed a ‘psycho-ethnography of the self’. Both auto-ethnography and psycho-biography tend to be narrative reflections of events interoceptively and/or psychically experienced. As such, neither have traditionally been subject to the scrutiny of formal ethics (Christians, 2011; Lapadat, 2017), as they embrace the epistemic rejection of empirical objectivity (Edwards, 2021; Poole, 2022), or indeed experience the rejection by ethical committees and institutional review boards who deem neither auto-ethnography nor psycho-biography forms of ‘research’, by their narrow definition (Tullis, 2013), but rather forms of inquiry achieved through the process of writing (Richardson, 1998; van Maanen, 2011). Furthermore, these approaches are ontologically different to mainstream research insofar as they have been described as a way or form of writing, and not analysis (Ellis, 2003; Neville-Jan, 2003; Elms, 2010), much in part to do with the spontaneity of their processes (Wilkinson and Wilkinson, 2018), the ‘analyst’ and the ‘subject’ being a singular entity (Denzin, 2003, 2006, 2014; Wilkinson, 2020), and the biographic nature causing an inability to create a dichotomy between self and other, as is the case with research analysts and participants (Denshire, 2014; Wilkinson and Wilkinson, 2023). It is therefore to be cognisant of Sparkes’ (2013; pp. 207) warning on the ethical issues and dilemmas associated with these forms of writing: “…*our stories are not our own. In the process of writing about ourselves, we also write about others. In this act we run the risk of making those we write about not only recognisable to others but recognisable to themselves*…” and so any reference to others within the article below have been anonymised by simply using an initial to demarcate individuals from one another.

Notes were made during the days immediately preceding and following the conference and sporadically during the course of it. My recording of the notes was deliberately not regular to prevent a forced opinion on aspects of the conference, and therefore notes were organic, irregular – erratic even – and written in a note pad, within the conference programme, on my phone as saved messages and in messages to other people, or on my laptop as a ‘sticky note’, or on the back of pieces of paper I had picked up at the conference, or found at the bottom of my satchel (see Winkler, 2018). Notes were then collated, transferred into a word document transcribed with their dates and times assigned, and iteratively sorted and organised into broad thematic reflections of the event.

### 2.1 Positionality

Engaging in this form of reflexive practice is at once self-indulgent, but also sets the scene for vulnerabilities to show (Behar, 1996). This is especially true when one positions themselves at the centre of their own reflection and compares oneself – as I intend to in this article – to the other actors in the given setting, which in this context is the PoWES annual conference. There are, however, other attributes of positionality which should be addressed – the privileges I hold, and my position as a researcher of my own subjective experiences.

To start with positionality and privilege: I am a white, heterosexual, cis-gendered male, who is able bodied, and from a relatively comfortable middle-class family. I have been afforded a good education at a Russell Group University, which both enabled me to study on the continent, and pursue further academic education. I have also been fortunate to have had continuous employment since graduating as a Psychologist, as a fixed-term researcher, and latterly in a substantive post. The identity as a Psychologist is again a privileged one. Though not a protected title in the United Kingdom (BPS, 2017), the term is often socially and culturally regulated (see Silverio, 2019) and is subject to certain societal expectations as discussed by Cordella et al., (2016); p. 102: “*Evidence in the literature indicates that psychology is regarded favorably. The public, however, appears somewhat confused about the role and functions of psychologists. This may impact upon the capacity of professionals to assist the wider community*…”. Similarly, being an academic affords privileges not only in earning potential, but in occupational classification (which if the 2010 ONS definition is used, places higher education professionals as category 1 out of 8). Though with this, comes a whole system of academic ranking whereby I am then further categorised as an early career researcher, who, at the time of undertaking this ‘psycho-ethnography of the self’, was twenty-five years old, without a doctoral degree, and therefore also without academic tenure. I was – at the time of writing this article – therefore affected by the precarity of short-term/fixed-term academic contracts, coupled with the frustration associated with attempting to source funding for my doctoral research required to attain a PhD and the coveted title of ‘Doctor’. By the time of submission of this article, some three-to-four years later, I have secured said funding and achieved a substantive academic post. Naturally, I cannot not be the things I am, and so as with all reflexivity it is more about being aware of those factors and how they may influence, cause, or hinder certain experiences or relationships, rather than endeavour to change them (see also Wilkinson C. 2016; Wilkinson S. 2019).

Being a researcher as part of The British Psychological Society’s Psychology of Women and Equalities network is also worth reflecting on. I am after all a male researcher who works almost exclusively within the realm of women’s mental health and psychological wellbeing. My position is often in gendered opposition to the participants I engage in my research, but also to the colleagues with whom I work and – especially important for this article – the majority of members and attendees of the PoWES network and annual conference. This position as a male researcher in women’s studies research is not uncommon, and has been discussed by previous scholars (Hearn, 2008; David, 2017; Precopio and Ramsey, 2017). This is also something on which I have received brilliant mentorship and sound guidance. This mentorship has led to a series of writings on the topic of the self in my own research praxis (see Silverio, 2018a,b,c,d,e, 2021). Again, the awareness of this position has been fruitful for me to realise the strengths and limitations of me myself undertaking the work I do, and also has offered perspective and, perhaps most importantly, the time to be reflexive about work I have done, and work I have plans or aspirations to undertake.

## 3 Psycho-ethnography of the self

The reflections contained within, were made during the 2019 conference of The British Psychological Society Psychology of Women and Equalities Section, which is hosted annually during the second week of July, at Cumberland Lodge, situated in Windsor Great Park (though this has not always been the venue, and nor may it remain the venue for future PoWES conferences). Most delegates stay for the duration of the conference (three days, two nights), meaning for a small epoch of time, a community of feminist Psychologists (and other Social Scientists) spend not only the conference, but their mealtimes together, often whiling away the evenings into the small hours of the warm Summer.

### 3.1 Scholarship

PoWES is, fundamentally, an academic network, and the annual conference is a showcase of its members’ research work. Variety is not unusual for a PoWES conference, but it struck me this year that health and health-related psychological research was much more prominent in this year’s (2019) line-up:

It’s ironic – the year I present more social psychological research, is the year when health seems to have taken over the programme – there are parallel sessions on: ‘Health & Bodies’; ‘Sex & Sexualities’; and an entire session dedicated to ‘Pregnancy’ which I am really interested in seeing.

(Note made on 1^st^ Day, during Registration)

The intersection between health and critical psychology is a familiar one at PoWES, and one which I comfortably straddle having a critical approach to my work, and having always worked in health departments. The prospect of collaborating on new projects is always exciting, but this year, the prospect of collaborating with fellow ‘PoWES-ers’ (PoWES members) was especially exciting:

Super excited about the proposition of a symposium next year with T [delegate] and M [delegate]. This is what PoWES is all about for sure – coming here, discussing your work, finding people working on similar things, and then doing great research together – and most importantly, being excited about it!

(Note made on 3^rd^ Day, after a Coffee Break)

Scholarship in the areas of women’s health and feminist psychology tend to employ a qualitative approach and so there is often much discussion about methodology and methodological technique at the conference. There was an interesting change in how I perceived myself as a methodologist this year – one which I documented after a conversation with a PhD student from The U.S.A. who was attending the conference for the first time:

When did I switch to being the one who offered advice? I’m the one who often asks for it! It was good to talk to E [delegate] about how to apply Grounded Theory to their work. The data sounds like it will be really interesting – I wonder if I can get her on board with me and C [a Master’s student of mine – not present at the conference] and H [a colleague – not present at the conference] to work out how we might formalise this ‘feminist’ grounded theory methodology… Must remember to e-mail them all when back at work!

(Note made on 3^rd^ Day, after the Lunch Break)

The idea of not being at work, when attending a conference on behalf of my employer was interesting, though did not occur repeatedly. There is indeed more investigation needed in this area, however the rest of the theme of scholarship not only explored how I was experiencing my own position within the conference delegation, but also about the scholarship I went to listen to. These included talks I went to see by new delegates to **#PoWESconf** who are working on similar areas as I am:

A’s [delegate] approach to constructing femininity has made me think. I wonder if her work on body image would be appropriate for the book [I was due to propose] – I’d certainly like to include work on body image – this could work. *Remember to chase this when back*

(Note made on 1^st^ Day, during Parallel Session on ‘Feminism & Media’ in which I was also a speaker)

This feeling also covered the work I saw by colleagues or those people I knew from previous years at **#PoWESconf** and these were usually attended as a sign of support (as well as interest) to these speakers as they delivered their research to the PoWES audiences:

I can’t believe this is the first time I have heard T [delegate] talk! We’ve been at a couple of different conferences together, but always missed their sessions. The work on perinatal mental health services was really methodologically interesting and the results are so important – must tell L [a colleague – not present at the conference] about the findings.

(Note made on 3^rd^ Day, during Parallel Session on ‘Pregnancy’)

The theme of Scholarship also contained reflections of the conference as a whole:

The keynotes! Ah they were just fantastic. It was great to see M [previous PoWES Chair] do hers, so powerful, so emotionally charged, and it really resonated with my family’s experience as immigrants. And then K’s [previous PoWES Chair]. Well where do I start? There was so much learning in that hour. I have a lot to go away and read – lots that I realise I don’t know and new ways of approaching qualitative feminist research. It is definitely these keynotes which provide us ECRs the guiding light for our future in research.

(Note made on 3^rd^ Day, during the Q&A of the final Keynote of the conference)

The talks today were just superb – for the Friday, they were especially strong – which is not unusual for the conference as a whole, but stands out compared to Friday being the day most of us are a little worse for wear after the long day and late night of the Thursday.

(Note made on 3^rd^ Day, just before leaving)

These reflections were placed under the theme of ‘Scholarship’ as they were labelled relating to research, education, and the work of academics, when the notes I had made were subsequently reviewed. This theme contained reflections relating to ways of doing academic work and later presenting it at conferences whilst also suggesting how conferences can be spaces for scholarship to transform into both active and incidental mentorship – for example with the presentation of keynote talks being points of learning for all delegates from those who have been part of the (PoWES) academic community for longer. Scholarship was identified as a major theme in this ‘psycho-ethnography of the self’, as the act of personal scholarship – both learning from more experienced delegates, and in turn also offering advice to new(er) delegates – was pertinent throughout the notes I made during the time I was at the 2019 BPS PoWES annual conference.

### 3.2 Feminist praxis

Iterative reviewing and re-organising of notes made whilst at **#PoWESconf** generated a second theme of ‘Feminist Praxis’. These reflections were derived from observations of the practices PoWES delegates undertook during the conference. The shared understanding and mentorship which is so often lacking in academia, is on the contrary, strongly prevalent within the PoWES community, demonstrated by the following note made after a discussion of which I was a part on two of the outdoor benches, and which went on late into the first night:

“It seems that everyone has had a really long year this year. There was a sort of deflated-ness in the air this evening with all of us sharing a few struggles we have had since the last PoWES. It seems as if everyone just needs to get it out of their system tonight and I’m sure we’ll all be on form tomorrow.”

(Note made on the 1^st^ day, after returning to my room for the night)

The reflections encapsulated in this theme truely showed how flat the hierarchy feels at this conference. The shared support from late night conversations as seen above, continued over mealtimes, during coffee breaks, and at the wine receptions, and also in pockets of delegates who form little break-out rooms to discuss their work together in the grounds or in one of any number of sofas at Cumberland Lodge. The main thing about this, is that everyone’s voice is heard and valued:

“It’s funny how the boundaries of seniority just seem to melt away at PoWES – we all know who the Profs are, and we recognise who are ECRs, but in reality, the advice goes both ways – not just ‘top down’, but also ‘bottom up’ and ‘sideways’ too.”

(Note made on 2^nd^ day, before Lunch)

This idea of advice not simply filtering from those with more years of academia ‘under their belt’ to early career researchers is a particularly favourite aspect of **#PoWESconf** for me. Here the quotation is tapping into the fact that all delegates’ experiences are heard and valued, and therefore shared as communal strategies for navigating academia and negotiating particular issues faced. This idea was extended in the theme of Feminist Praxis with the idea of being comfortable in the knowledge that returning colleagues use **#PoWESconf** to continue to mentor and nurture one another, regardless of their experience, with the overwhelming feeling being that of wanting everyone to succeed:

“It’s good to know that when you need advice, the most senior PoWES-ers – the Profs, the Readers, the Heads of Department – those who have been around a long time and have seen it all – they are there for us and their advice is always so considered. I know R [delegate; Professor] and P [delegate; Professor] have always been there for me when I’ve needed it and I’ve seen a few conversations today that make me think Professorial advice mode is in full swing.”

(Note made on the 2^nd^ day, after returning to my room for the night)

Overall, the theme of Feminist Praxis in my notes made over the three days of **#PoWESconf** can be summarised as not only one of the highlights of my academic calendar, but also an event which is important in grounding me as an academic.

“This conference is a lifeline in the clamour of academia which I can ill afford to miss. It gives me the time to re-set, the impetus to go again, and the grounding to know I am not alone in the fight for critical research.”

(Note made on the 3^rd^ day, having returned home)

When organised, these reflections were coded in relation to mentorship, collegial relationships, and community – all lensed using a feminist viewpoint – and thus could be grouped under the theme of ‘Feminist Praxis’. It is evident amongst these reflections that the way in which delegates interact at **#PoWESconf**is in a supportive manner to achieve the ultimate goal of academic success for us all. Rather than interaction occurring according to hierarchy and deference to those more senior, there is esteem and reverence for all colleagues from all levels of experience, from all institutions, and from all corners of the Earth. Finally, this theme concentrated on the mentorship which was present at the 2019 conference (and which I have always noted since first attending) and how not only was the conference a place in which academics can seek and provide advice, but that the place itself and the way the conference is set up, facilitates those mentoring conversations to occur.

### 3.3 (Safe) academic spaces

Space was especially important as the space has to be correct for researchers of all levels to feel confident in discussing their academic troubles, concerns, worries, successes, failures, pride, and ambition:

“This space [the PoWES annual conference] is exactly what academia should be about – a space for shared learning, for academics to engage in interesting conversation, and to feel safe in having those difficult conversations about their struggles.”

(Note made on 1^st^ day, shortly after arrival)

Likewise, it had to be ‘safe’ enough so delegates could be daring with what they presented and challenge normative ways of thinking about states of being. This was summed up in the following excerpt, where I found a talk I wanted to attend because of the wonderfully contentious title:

“Mad mothering” – what a title!… I’m definitely going to that! I love the fact that we can be challenging, critical, daring even at this conference – to come out with bold titles, and difficult findings to tackle the damaging dominant discourses head on. PoWES always provides that safe space where we can discuss the uncomfortable and the hard to hear and know that we will be received warmly and with encouragement to go forth and make changes, positive changes for those we research. This is really the point of academia – making those changes and challenging the outdated or dangerous discourses which exist to – knowingly or not – supress and control. I am excited to see what the team will present.

(Note made on 2^nd^ Day, Early Morning – whilst perusing the programme over a coffee in my room before breakfast)

Amongst my notes for this theme, one reflection captured in this ‘psycho-ethnography of the self’ was how space at the conference can not only be taken up in person, but also virtually, by the number of Tweets delegates are putting out. The space I consumed at the physical conference and the **#PoWESconf** virtual space I could monitor when not physically present at talks, thus became an important factor. It was my presence at – or in actual fact, my absence from – the conference which made for some of the most compelling reflections. At **#PoWESconf** it is possible to excuse one’s self from the proceedings, and therefore I could *be* physically absent, but keep updated on what was going on during these hours by following the **#PoWESconf** Twitter stream, and therefore *feel* present. Not only that, the act of removing myself from the conference for a few hours, showed some growth in my confidence at **#PoWESconf** and within the PoWES community – for the first time, I no longer felt I had to attend something during every session, but I could quietly, and without making excuses, disappear and re-appear as I pleased, being content that I was fulfilling my duty as a conference delegate, and quieting the voice in the back of my mind which was repeatedly rehearsing a list of jobs I had to do for work and other collaborations:

“Today was the first time in the five years I have been coming to PoWES that I felt fine – as in really okay – with taking some time away from the talks to do some writing. The ability to slip off and ‘keep the day job going’ free of judgement for missing some of what we all came here for [the conference talks] is most definitely welcomed. Also, to be able to write – completely uninterrupted – in such a beautiful setting works wonders for the brain to focus, and really entrench itself in what has been, and what still needs to be written!”

(Note made on the 2^nd^ day, whilst working in my room)

One of my final reflections summed up how Cumberland Lodge works as a space for enabling everything that is good academically and psychologically about **#PoWESconf**:

“Every year it becomes easier to come to this place, but harder and harder to leave.”

(Note made on 3^rd^ day, shortly before leaving)

Having written this ‘psycho-ethnography of the self’, it became increasingly apparent a central thread through all the themes was one of space, and perhaps most importantly ‘safe’ spaces in academia. Throughout I had coded for my own awareness of the space which I inhabited and/or took up amongst what could be seen as three (imagined) spaces of the delegation, setting, and community. Seeking advice on the original draft manuscript, valued and sage colleagues provided excellent counsel by suggesting the idea of space may require some ‘explicit acknowledgement’. And so, as with much qualitative work, I began to iteratively look at my notes. Space did indeed, become an important theme in its own right.

### 3.4 Positioning the self

The final theme presented here is the largest, and perhaps the most important for this method of ‘psycho-ethnography of the self’. This theme focuses on my reflection and reflexivity of the position at **#PoWESconf**, my actions there, and my interaction with other delegates and the conference itself.

“I am really excited to see everyone – I hope all the usual crowd make it this year – it would be good to catch up with a few people I haven’t seen for a year or two.”

(Note made on the 1^st^ day, before leaving home to drive to Windsor)

Unfortunately, there were a few of the ‘usual crowd’ missing and one of my early reflections demonstrates a disappointment that I would not get to see some of those people who first welcomed me five years ago to PoWES, whilst simultaneously enlightening me to a transition I had made with the fact that I now felt secure enough (as an academic and a **#PoWESconf** delegate) to be at the conference without those figures there to lean on as a support.

“There’s a few key faces missing this year which is a shame – [list of approximately 10 PoWES-ers not present at the 2019 **#PoWESconf**] just off the top of my head, and there is definitely still a core group of us here. Funny!… When did it become ‘us’? This year definitely feels like I’m one of the furniture – the old PoWES stock who are here every year. Not quite sure when that happened, but I like it.”

(Note made on the 1^st^ day, after Dinner)

Whilst missing PoWES-ers were noted in the journal I kept; the acknowledgement of new delegates was also captured. It also became a point of reflection on the time when I had once been wide-eyed with a mix of awe and slight terror at the prospect of attending this conference, to unknowingly accepting a transition to a more established member, who was comfortable at the conference and amongst the conference delegates. This was most eloquently outlined in the following quotation whereby I position myself as no longer a ‘new face’ within the PoWES community:

“There are lots of new faces this year… I’m not entirely sure when I became one of the old ones.”

(Note made on the 2^nd^ day, after Lunch)

As well as recording my reflections on the delegates and my interaction with both the new and the old faces of PoWES, my notes reflected the fact that PoWES has a thriving on-line community between our Facebook pages and Twitter handle. I reflected on how this virtual channel at the conference can be utilised, to make **#PoWESconf** ‘trend’ on Twitter, but also to share highlights of talks in one parallel session to other delegates who are attending a different parallel session – something Greenhow et al. (2019) refer to as ‘conference backchannelling’. When looking at my own Twitter feed, those days in July when the conference takes place always demonstrates a peak of activity for me:

“People must think I am a mad-man liking and re-tweeting almost everything on the **#PoWESconf** stream – but I really want to make sure our messages are getting out there. We haven’t trended in a couple of years, so I think all the tweeters are making sure people know we have started!”

(Note made on the 1^st^ day, after the first Parallel Session had finished)

The PoWES community – though concentrated amongst some Universities, is generally spread out across many institutions, and across various countries. Whilst Twitter allows all delegates to see what is being presented at other parts of **#PoWESconf** which we are not attending, the extension of the PoWES community can be seen to continue in this virtual manner even after the conference has taken place:

“The PoWES conversation seems to be continuing on Twitter after the conference much more than I remember from previous years. It’s actually quite nice to keep the dialogue going between PoWES-ers, congratulating one another on publications, sharing ideas for new research and meet-ups to write, and even if it is just silly things like wishing one another a restful and relaxing (and dare I say it… work free) holiday!”

(Note made some days after the 2019 **#PoWESconf** ended)

Within the theme of ‘Positioning the Self’ reflections focused on how I positioned myself within the conference delegation, within the conference setting, and within the PoWES academic community. My position as a male researcher in itself is unique, as there are only a few who regularly attend, but in all, this theme presents a clear understanding of my established *self* and my developing *role* at the PoWES conferences and amongst the PoWES community. This theme demonstrates the growth I have achieved and the re-positioning of myself as no longer a ‘new face’ to the conference, but as someone who is now becoming one of the ‘usual crowd’ and who can share my experiences – both within and outside PoWES – with those who are new to the PoWES community and/or are attending a **#PoWESconf** for the first time.

## 4 Discussion

In this article, I present a ‘psycho-ethnography of the self’, using notes made during the 2019 annual conference [**#PoWESconf**] – the fifth one I had attended – using methodologies abstracted from the fields of auto-ethnography and psycho-biography. The ‘psycho-ethnography of the self’, as I have referred to it, rendered four themes of: ‘Scholarship’, ‘Feminist Praxis’; ‘(Safe) Academic Spaces’; and ‘Positioning the Self’.

The first of these themes suggests conferences, and more specifically, this particular conference, acts as a place of shared learning between me and those more established academics and also between me and those attending **#PoWESconf** for the first time. In doing so, ‘Scholarship’ is seen to extend from institution to institution and therefore country to country in a supportive and collegiate way, enabling new collaborations and opening the possibility for forming strong working relations and inevitably sound friendships borne out of those professional relationships.

The second theme – ‘Feminist Praxis’ – demonstrates the **#PoWESconf** as a space for garnering advice on academic careers and (re-)energizing one another for the next academic year ahead. Here, my reflection is that I have developed as an academic, as a researcher, and as a scholar having attended this conference each year for the last five years at the time of writing (nine years in total by time of manuscript acceptance) and am now able to act as part of that support network for my peers, friends, and colleagues.

Originally, ‘space’ was viewed to be important in all themes, but on reflection, ‘(Safe) Academic Spaces’ was identified as a theme in its own right. The (psycho-ethnographic) notes I kept addressed the space which I perceived I and others occupied at **#PoWESconf** physically and virtually and my awareness of my own occupation of space became a point for reflection and reflexive practice upon my own positioning within the PoWES community and the conference. This also interrogated the importance of the physical space (Cumberland Lodge) and how that enabled the community to enact feminist praxes within academia.

Finally, this ‘psycho-ethnography of the self’ of the 2019 conference generated a fourth theme addressing ‘Positioning the Self’ whereby my position at the conference was interrogated with relation to how it has changed as a returning delegate. This has documented and demonstrated the perception I have of my changing role within the PoWES community from a ‘new face’ to an ‘old one’.

Suggested lessons from this ‘psycho-ethnography of the self’ would be for conference organisers to ensure there is an egalitarian and equitable feel about the conference and not under-estimated how daunted some delegates may be. Findings also suggest that the space in which conferences are held are vitally important, and those organising conferences should think carefully about their venue. For delegates, the advice is to keep attending. The connections made at conferences are invaluable, but make sure you are attending the ‘right’ conferences for you and your research. Furthermore, and finally, do not be afraid of stepping out and attending to the other aspects of your personal and work lives which need maintenance. Conferences should be spaces of learning and sharing knowledge and where possible advancing it, but everyone in attendance will have left something on the backburner whilst they are there. Ostensibly, we are all in the same busy ‘boat’, but some may be weathering choppier waters, making conference attendance anything from a much anticipated positive interlude in one’s working life, to a business engagement, to an escape.

In summary, The British Psychological Society’s Psychology of Women and Equalities Section annual conference acts as the annual meet-up for feminist scholars working in the fields of gender, sex, and sexuality from all over the United Kingdom and indeed, from other countries and continents. This conference acts as a ‘safe space’ to share new research, seek advice and guidance on academic work and scholarship, and also reinvigorate researchers on their journeys, no matter what level of experience scholars have.

## 5 Concluding commentary

Academic conferences have been discussed as – and continue to be – places where researchers, practitioners, and students can cross the institutional and sometimes also the disciplinary divides to meet and establish good working (and occasionally also social) relationships with fellow scholars working on similar research as themselves. In doing so, scholars can temporarily leave their academic ‘homes’ and travel to a central place in order to meet their contemporaries from across the globe. My role at this conference has, on reflection, changed – and my growth has been evidenced through my reflexivity. The British Psychological Society’s Psychology of Women and Equalities Section annual conference continues to be a place of great scholarship and I continue to learn from other delegates – both new and old – in a shared endeavour to place Feminist Psychology firmly on the map of Psychology, globally, whilst working to make the lives of our participants, clients, and patients, better, safer, and more equitable. For these reasons, I can see this conference being an annual and non-negotiable event in the calendar for more than just the foreseeable future and would implore others – established academics and new ones – to do the same.

## 6 Afterword

The keen amongst readers will realise this article comes some time after it was first written. The truth of the matter is that shortly after completion, the pandemic descended and it felt terribly indulgent to put such a self-reflective piece into the world, when most research efforts – including my own – were being channeled towards understanding the effects of the global health crisis. Now that the pandemic itself is behind us (albeit not the effects which we expect to be long-lasting), I hope readers find this an acceptable time for this article to be introduced into the literature-base.

## Data availability statement

The original contributions presented in the study are included in the article, further inquiries can be directed to the corresponding author.

## Author contributions

SAS: Conceptualization, Data curation, Formal analysis, Investigation, Methodology, Project administration, Resources, Validation, Visualization, Writing – original draft, Writing – review & editing.
